# Microcirculatory impact of vatinoxan and fentanyl in male Wistar rats sedated with medetomidine and midazolam

**DOI:** 10.1186/s12917-026-05483-y

**Published:** 2026-04-21

**Authors:** Emily Lindh, Anna Meller, Karoliina Alm, Marja Raekallio, Juhana Honkavaara

**Affiliations:** 1 Faculty of Veterinary Medicine, Department of Equine and Small Animal Medicine, University of Helsinki, Helsinki, Finland; 2 HiLIFE Institute, Laboratory Animal Centre, University of Helsinki, Helsinki, Finland

**Keywords:** Medetomidine, Vatinoxan, Midazolam, Fentanyl, Microcirculation, Rats, Cardiovascular

## Abstract

**Background:**

Alpha2-adrenoceptor agonists, such as medetomidine, are pivotal drugs in laboratory rodent sedation and anesthesia. However, they induce marked systemic cardiovascular adverse effects, which can be mitigated with vatinoxan, a peripherally acting alpha2-adrenoceptor antagonist. We investigated the impact of vatinoxan (5 mg/kg) and fentanyl (0.010 mg/kg), an opioid-receptor agonist, on cutaneous microcirculation in male Wistar rats sedated with medetomidine (0.25 mg/kg) and midazolam (2.0 mg/kg). Mean arterial blood pressure (MAP) and pulse rate (PR) were measured. Cutaneous microcirculation was assessed with simultaneous quantification of laser-Doppler flow (LDF) and tissue hemoglobin saturation (T-HbO2). Data were analyzed with Dunn’s tests, Student’s t-tests and Spearman’s correlation tests with Bonferroni-adjusted alpha-levels when appropriate.

**Results:**

Overall median [range] LDF (139 [95–561] vs. 120 [72–201] perfusion units) and T-HbO2 (57 [39–75] vs. 41 [18–75] %) from pooled data including all time points were significantly higher (*p* < 0.001) for the treatments with vatinoxan. Similarily, pooled MAP was lower with vatinoxan (92 ± 14 mmHg [mean ± SD]) than without it (139 ± 18 mmHg) (*p* < 0.001). Furthermore, MAP was moderately negatively correlated with LDF (Spearman’s rho = − 0.439, *p* < 0.001). Pooled pulse rates were also significantly higher with the addition of vatinoxan (323 ± 33 bpm) compared with treatments without it (281 ± 29 bpm) (*p* < 0.001). Fentanyl did not significantly alter any of the outcomes.

**Conclusions:**

The addition of vatinoxan alleviated the hypertension and improved pulse rate, cutaneous microcirculation and tissue oxygenation in male Wistar rats sedated with these medetomidine-based protocols. By reducing the peripheral adverse effects attributed to medetomidine, vatinoxan may contribute to refinement of laboratory rodent sedation.

**Supplementary Information:**

The online version contains supplementary material available at 10.1186/s12917-026-05483-y.

## Background

 Alpha2-adrenoceptor agonists (α_2_-agonists), such as xylazine, medetomidine and dexmedetomidine, are widely used for sedation and anesthetic-sparing in laboratory rats [1–4]. In addition to their central sedative and antinociceptive effects [5], these compounds stimulate peripheral α_2_-adrenoceptors. Activation of α_2_-adrenoceptors in vascular smooth muscle leads to vasoconstriction and a rapid increase in arterial blood pressure, followed by reflex bradycardia [6, 7]. A significant decrease in cardiac output (CO) is observed after therapeutic doses in many species [8–13]. In dogs, the reduced heart rate is closely associated with decreased CO and oxygen delivery (DO_2_) along with increased tissue oxygen extraction ratio, reflecting inadequate oxygen supply after medetomidine administration [14]. However, studies on real-time assessment of microcirculation and oxygenation during α_2_-agonist sedation remain sparse and only a few reports have described similar non-invasive methods of assessing cutaneous microcirculation after α_2_-agonist administration in rodents [15, 16]. In a previous study assessing laser Doppler flow in medetomidine-sedated dogs, significant reduction intestinal and skeletal microcirculation was observed [17]. Furthermore, in dogs treated with dexmedetomidine, the buccal mucosal microcirculation assessed by side-stream dark field imaging was reduced when compared with acepromazine, another sedative with a vasodilatory action [18]. Similarly in mice, skin microcirculation was significantly reduced by dexmedetomidine compared with acepromazine, when assessed by laser Doppler perfusion imaging [16].

To counteract the peripheral cardiovascular effects of α_2_-agonists, vatinoxan (also known as MK-467 or L659,066) has been investigated in various mammalian species, including mice and rats [19–21]. Vatinoxan is a hydrophilic α_2_-adrenoceptor antagonist that does not readily cross the blood-brain barrier [22] and therefore it does not interfere with sedation [19, 20]. Furthermore, by attenuating the α_2_-agonist induced vascular effects, vatinoxan increased oral mucosal microcirculation in dogs treated with medetomidine or a combination of medetomidine and methadone, a commonly used opioid-receptor agonist [23, 24]. A preformulated mixture containing both medetomidine and vatinoxan is commercially available in the U.S. and European markets and is approved for intramuscular sedation in dogs for short procedures.

In veterinary research, a novel hybrid technology combining laser Doppler flowmetry (LDF) and diffuse reflectance spectrometry (DRS) has been previously applied in dogs, to assess both oral mucosal and testicular surface microcirculation [24]. To the authors’ knowledge, its application in rodents has not been reported.The technology produces dynamic measurement of microcirculation in absolute units [25, 26].DRS uses visible to near-infrared white light to quantify tissue hemoglobin oxygen saturation (T-HbO₂) and the concentration of red blood cells (RBC tissue fraction). In addition, the technology separates flow velocities providing speed-resolved perfusion, where lower flow velocities (< 1 mm/s) are associated with nutritional, capillary perfusion, intermediate velocities (1–10 mm/s) correspond to venular and/or small arteriolar flow, and higher velocities (> 10 mm/s) reflect transportational flow in larger arterioles and vessels within the sampled area [25–29]. The speed-resolved perfusion groups are expressed as the product of the number of red blood cells and the flow speed, respectively, and summed together to provide total perfusion. The primary aim of this study was to investigate the effects of vatinoxan on cutaneous microcirculation in rats sedated with medetomidine-based protocols. We further aimed to assess whether cutaneous microcirculation is associated with non-invasive mean arterial pressure (MAP) and/or pulse rate (PR) in male Wistar rats sedated with medetomidine and midazolam. The additional impact of fentanyl was also investigated. We hypothesized that cutaneous microcirculation and oxygenation would be higher in rats receiving vatinoxan with medetomidine-based protocols. Additionally, we hypothesized that the addition of fentanyl would further decrease PR and arterial hemoglobin oxygen saturation (SpO_2_) in rats breathing room air [29], without significantly affecting cutaneous microcirculation.

## Methods

This prospective, experimental terminal study utilized 32, healthy male Wistar rats. The animals were purpose-bred and acquired from Envigo RMS B.V., Netherlands, aged 10 weeks and weighing 339–410 g. The study was approved by the Project Authorisation Board of the Regional State Administrative Agency in Finland, license number ESAVI/39,801/2023. The license complies with European Union legislation and adheres to the requirements of the Animal Research: Reporting of In Vivo Experiment guidelines (ARRIVE 2.0) [30]. Rats were housed in pairs in individually ventilated cages (Blue Line 1500U for Rats, Tecniplast, Italy) and controlled specific pathogen free facilities at the University of Helsinki’s Laboratory Animal Centre, which were maintained under regulated temperature (21 °C ± 1 °C), humidity (55% ± 10%), and 12/12 light/dark cycles with lights turned on at 6.00 a.m. They were fed ad libitum a standard rodent diet (Teklad Rodent Dier 8460, Inotiv, IN, USA) with ad libitum access to purified water.

The rats were randomly assigned to one of four treatment groups (*N* = 8 per group) (Table 1.)

**Table 1:** Study protocols. All drugs were administered SC in the same syringe. ^a^ Dorbene 1 mg/ml, Laboratorios Syva S.A, Spain, ^b^ Midazolam Hameln 5 mg/ml, Hameln Pharma GmbH, Germany, ^c^ Fentanyl Hameln 0.05 mg/ml, Hameln Pharma GmbH, Germany, ^d^ Zenalpha 0.5/10 mg/ml (medetomidine HCl 0.5 mg/ml + vatinoxan HCl 10 mg/ml), Apotek Produktion & Laboratorier AB, Sweden

Group	medetomidine HCl (mg/kg) ^a/d^	midazolam HCl (mg/kg) ^b^	fentanyl citrate (mg/kg) ^c^	vatinoxan HCl (mg/kg) ^d^
MM	0.25	2.0		
MMV	0.25	2.0		5.0
MMF	0.25	2.0	0.01	
MMFV	0.25	2.0	0.01	5.0

Dosages were derived from previous studies and were expected to produce a reliable level of sedation [2, 20, 31]. Drugs were pre-mixed each day prior to the trials and diluted with saline to an equal dose volume of 1.1 ml/kg.

The rats were weighed with a dynamic precision scale (Mettler Toledo JE3200, Mettler-Toledo, LLC., OH, USA) before administering the treatments subcutaneously (SC) in the caudal section of the flank. Drugs were delivered in a single syringe and a 25 G 16 mm hypodermic needle, after which the rat was placed into an individual cage until cessation of spontaneous movement. After the righting reflex was lost, rats were laid by hand in dorsal recumbency on a heating pad (Far Infrared Surgical Warming Pad DCT-25, Kent Scientific Co. CO, USA) and bubble wrap placed on top of the animal. The rats were breathing room air spontaneously.

**Fig. 1:**
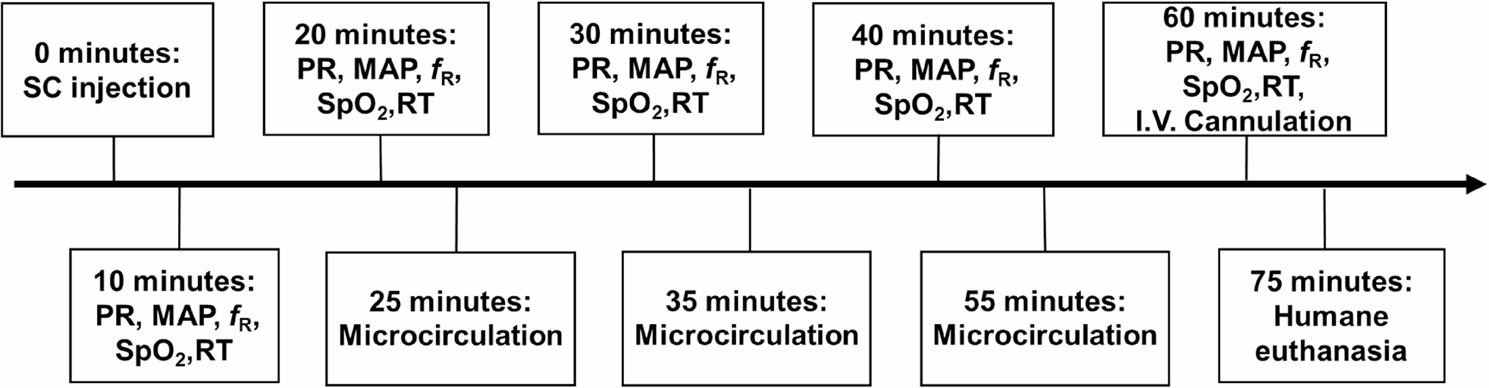
Study timeline. PR = pulse rate, MAP = mean arterial pressure, fR = respiratory rate, RT = rectal temperature

Figure 1 presents a detailed timeline of the study. Assessments were made by a single investigator (EL) who was masked to the treatment.

Non-invasive MAP (CODA Noninvasive Blood Pressure System, Kent Scientific Co. CO, USA) was measured from the tail, and PR and SpO_2_ (NONIN PalmSAT 2500 VET, MN, USA) from the right hind foot. Respiratory rate (ƒR) was assessed visually by counting chest movements, and rectal temperature (RT) via a handheld thermometer (Microtherma 2 Type T Thermometer, Agntho’s, Sweden).

Microcirculatory variables were measured with the PeriFlux EPOS 6500 system (Perimed, Järfälla, Sweden). The system consisted of a PF 6010 laser Doppler unit (including a laser light source at 785 nm and an optical passband filter at 785/40 nm), a PF 6060 spectroscopy unit, a broadband white light source (Ava-light-HAL-S, Avantes BV, The Netherlands), and a fibre-optic probe. The unit was calibrated and verified according to the manufacturer’s instructions before and during the study.

At 15 min post-injection, fur was clipped and shaved caudally from the level of the sternum to the navel to create a 2 cm × 2 cm measurement area. The fibre-optic probe was then secured on the skin using a double-sided adhesive ring (Double Stick Disc 2181, 3 M Co., MN, USA), ensuring no external pressure, pull, or torque was applied once attached. The same measurement site was used for all animals at all time points. The device was connected to a PC laptop running EPOS Manager software (EPOS Manager 2.2., Perimed AB, Sweden), enabling continuous real-time monitoring. Two-minute recordings were performed at three distinct time points, and analyses were based on averaged values from each time point. The variables recorded included LDF (in arbitrary perfusion units, PU), T-HbO_2_ (%), RBC tissue fraction (%), total perfusion (% RBC × mm/s) with speed-resolved perfusion into three velocity ranges (< 1 mm/s, 1–10 mm/s, and > 10 mm/s, expressed as % RBC × mm/s).

After 60 min, the rats were cannulated into the tail vein (24 G 19 mm Vasofix Certo, B.Braun, Melsungen AG, Germany or 26 G 19 mm Terumo Versatus-W, Terumo, Japan), and at 75 min euthanized with 50 mg/kg pentobarbital (Euthoxin 400 mg/ml, Chanelle Pharmaceuticals Manufacturing Ltd., Ireland), diluted with saline to 1 ml, and followed by cervical dislocation.

### Statistical methods

With a power of 80% and an alpha-level set at 0.0125, eight animals were required to detect a generic 30% mean difference with a ± 15% standard deviation (SD) difference in LDF and/or a 50 ± 25 bpm difference in PR between treatments. The magnitudes of these effects were considered clinically relevant and expected to extend to all microcirculatory variables and MAP, too. Statistical Package for Social Sciences (SPSS version 29 0.2.0 IBM, NY, USA) was used for all statistical analyses. Data distribution was assessed using the Shapiro-Wilk test. For all microcirculation parameters, SpO₂ and fR, the non-parametric Kruskal-Wallis test was used for the effect of treatment, between MM and MMV, MMF and MMFV, and between MM and MMF and MMV and MMFV, followed by a post-hoc Dunn’s test for multiple pairwise comparisons where applicable. For the analysis of parametric data, MAP and PR, a repeated-measures analysis of variance was applied, followed by Bonferroni corrected independent samples t-test for pairwise comparisons between MM and MMV, MMF and MMFV, and between MM and MMF and MMV and MMFV. Spearman’s correlation analyses were performed to explore associations between PR, MAP, PR:MAP ratio and microcirculation [24], and SpO_2_ and T-HbO_2_. Results were considered statistically significant with an adjusted *p* < 0.05.

## Results

Thirty-one rats completed the study. One rat in the MMV group was excluded from the study due to a dosing error. All rats became clinically sedated and remained in dorsal recumbency throughout the observational period.

**Fig. 2:**
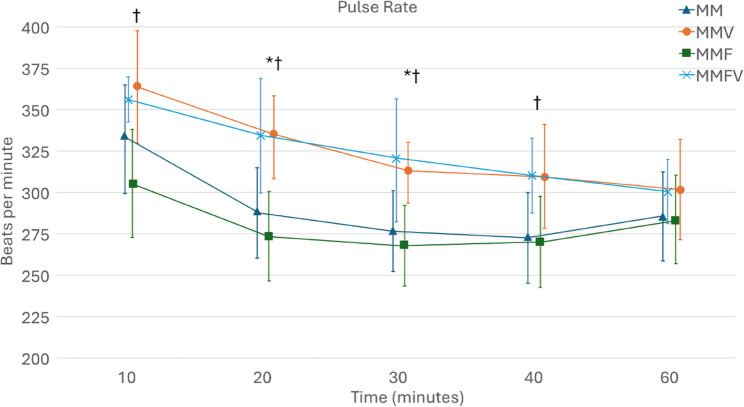
Pulse rates in Wistar rats administered with medetomidine HCl 0.25 mg/kg and midazolam HCl 2 mg/kg (MM) *N* = 8, MM  + vatinoxan HCl 5 mg/kg (MMV) *N* = 7, MM + fentanyl citrate 0.01 mg/kg (MMF) *N* = 8 or MMF + vatinoxan HCl 5 mg/kg (MMVF) *N* = 8. The data are presented as mean ± SD. * Significant difference at *p* < 0.05 between MM and MMV. † Significant difference at *p* < 0.01 between MMF and MMFV

**Fig. 3:**
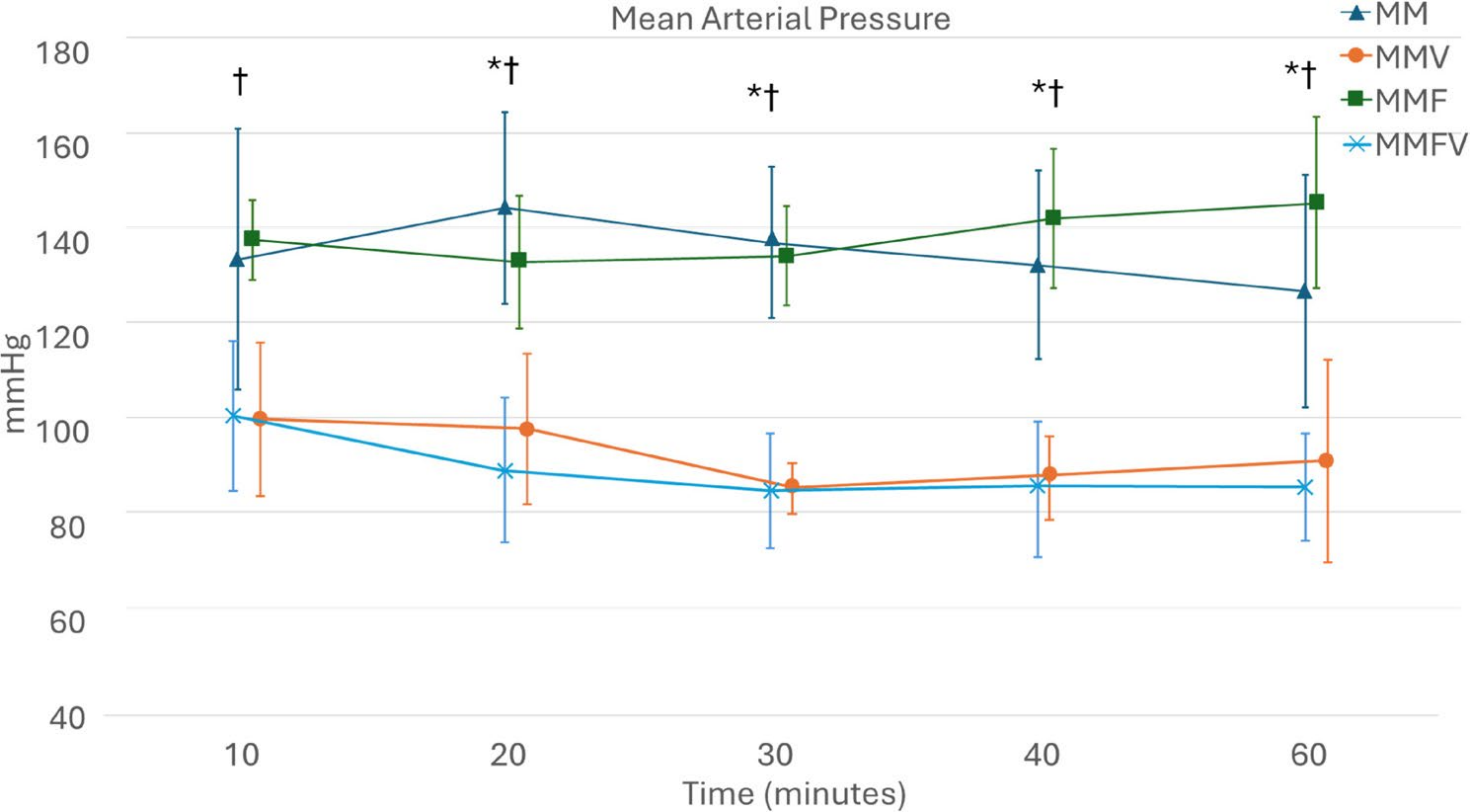
Mean arterial pressures in Wistar rats administered with medetomidine HCl 0.25 mg/kg and midazolam HCl 2 mg/kg (MM) *N* = 8, MM + vatinoxan HCl 5 mg/kg (MMV) *N* = 7, MM + fentanyl citrate 0.01 mg/kg (MMF) *N* = 8 or MMF + vatinoxan HCl 5 mg/kg (MMVF) *N* = 8. The data are presented as mean and ± SD. * Significant difference at *p* < 0.01 between MM and MMV. † Significant difference at *p* < 0.01 between MMF and MMFV

PR was significantly higher with MMVF at all time points compared with MMF for the first 40 min. With MMV, PR was significantly higher compared with MM from 20 to 40 min post-injection (Fig. 2.). MAP was significantly lower with vatinoxan treatments at all time points after 10 min (Fig. 3.). Fentanyl did not have a significant effect as no differences in PR or MAP were observed between MM and MMF or MMV and MMFV.

**Fig. 4:**
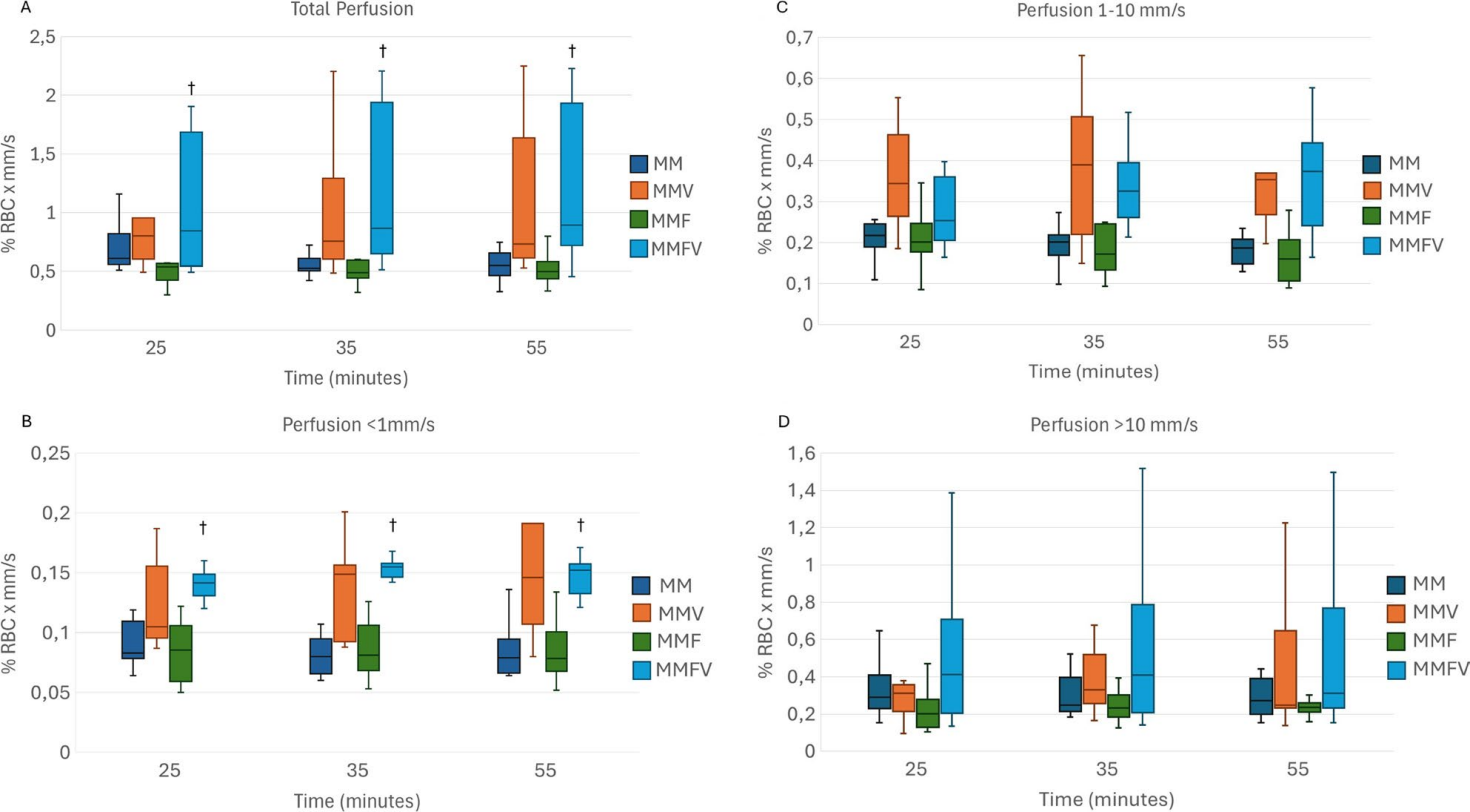
A-D Total perfusion (**A**) and speed-resolved flow at < 1 mm/s (**B**), 1–10 mm/s (**C**) and > 10 mm/s (**D**) in Wistar rats administered with medetomidine HCl 0.25 mg/kg and midazolam HCl 2 mg/kg (MM) *N* = 8, MM + vatinoxan HCl 5 mg/kg (MMV) *N* = 7, MM + fentanyl citrate 0.01 mg/kg (MMF) *N* = 8 or MMF + vatinoxan HCl 5 mg/kg (MMVF) *N* = 8. The data are presented as median and interquartile range (boxes) and range (whiskers). * Significant difference at *p* < 0.05 between MM and MMV. † Significant difference at *p* < 0.05 between MMF and MMFV

**Fig. 5:**
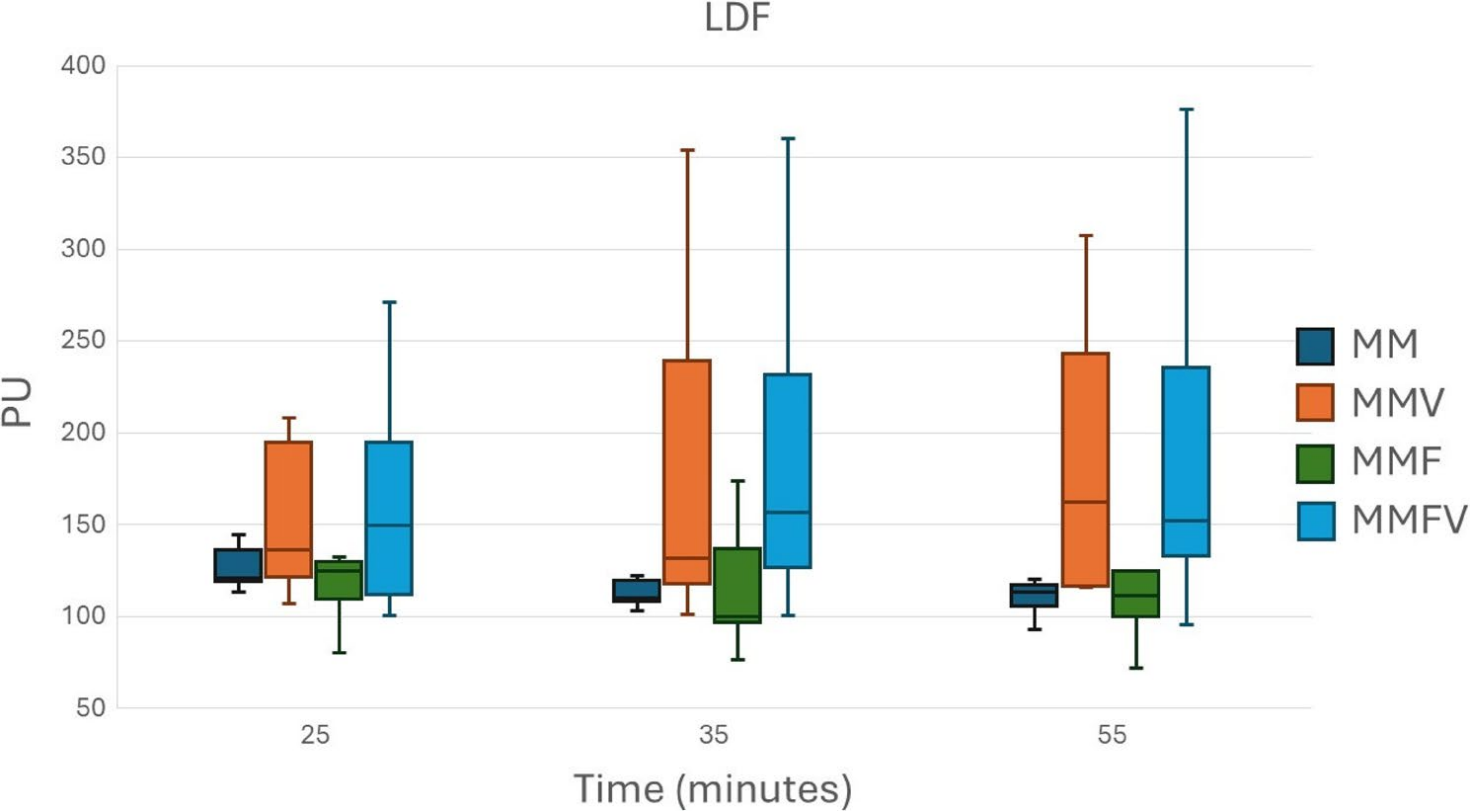
Laser-Doppler flow (LDF) in Wistar rats administered with medetomidine HCl 0.25 mg/kg and midazolam HCl 2 mg/kg (MM) *N* = 8, MM + vatinoxan HCl 5 mg/kg (MMV) *N* = 7, MM + fentanyl citrate 0.01 mg/kg (MMF) *N* = 8 or MMF + vatinoxan HCl 5 mg/kg (MMVF) *N* = 8. The data are presented as median and interquartile range (boxes) and range (whiskers). PU = perfusion units

**Fig. 6:**
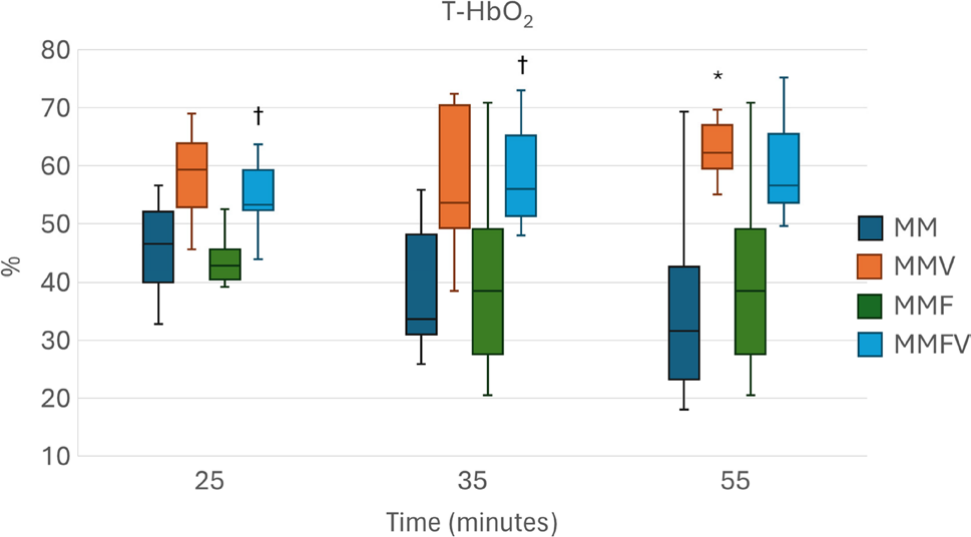
Tissue hemoglobin oxygen saturation (T-HbO_2_) in Wistar rats administered with medetomidine HCl 0.25 mg/kg and midazolam HCl 2 mg/kg (MM) *N* = 8, MM + vatinoxan HCl 5 mg/kg (MMV) *N* = 7, MM + fentanyl citrate 0.01 mg/kg (MMF) *N* = 8 or MMF + vatinoxan HCl 5 mg/kg (MMVF) *N* = 8. The data are presented as median and interquartile range (boxes) and range (whiskers). * Significant difference at *p* < 0.05 between MM and MMV. † Significant difference at *p* < 0.05 between MMF and MMFV

**Fig. 7:**
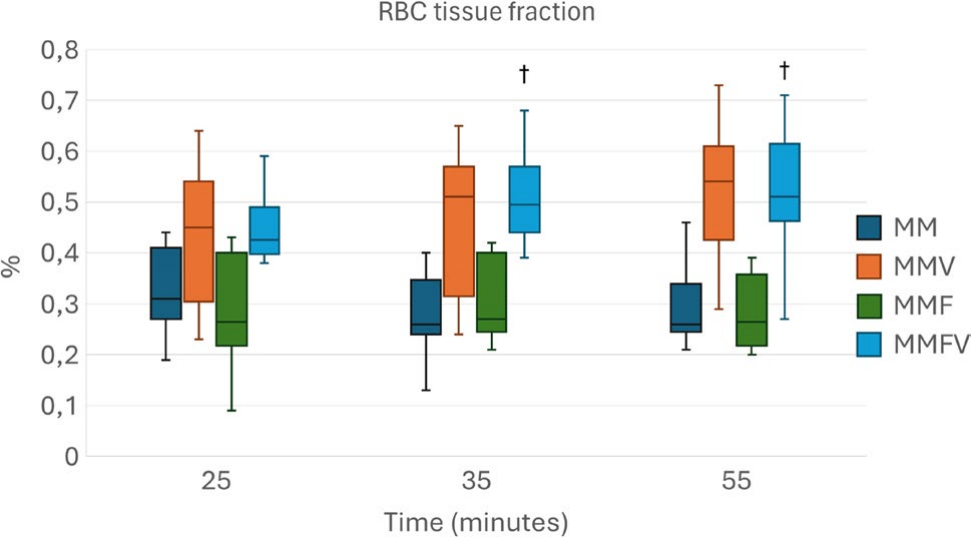
Concentration of red blood cells in tissue (RBC tissue fraction) in Wistar rats administered with medetomidine HCl 0.25 mg/kg and midazolam HCl 2 mg/kg (MM) *N* = 8, MM + vatinoxan HCl 5 mg/kg (MMV) *N* = 7, MM + fentanyl citrate 0.01 mg/kg (MMF) *N* = 8 or MMF + vatinoxan HCl 5 mg/kg (MMVF) *N* = 8. The data are presented as median and interquartile range (boxes) and range (whiskers). † Significant difference at *p* < 0.05 between MMF and MMFV

Overall, microcirculatory variables appeared to be higher with vatinoxan treatments, but statistical significance was reached only sporadically (Figs. 4, 5, 6 and 7).

The Spearman correlations between microcirculation, PR, and MAP are presented in Table 2. An exploratory PR: MAP ratio was calculated, which in most cases resulted in higher correlation coefficients between the PR: MAP ratio and microcirculatory parameters compared with PR or MAP alone (Table 2). The correlation between SpO_2_ and T-HbO_2_ was weak and not statistically significant (rho 0.199, *p* = 0.36).

**Table 2:** Spearman’s rank correlation coefficients (Spearman’s rho) and their respective p-values (in brackets) between PR, MAP, their ratio and microcirculatory parameters from pooled data of rats administered with medetomidine 0.25 mg/kg and midazolam HCl 2 mg/kg (MM) *N* = 8, MM + vatinoxan HCl 5 mg/kg (MMV) *N* = 7, MM + fentanyl citrate 0.01 mg/kg (MMF) *N* = 8 or MMF + vatinoxan HCl 5 mg/kg (MMVF) *N* = 8. PU = Perfusion units

	PR	MAP	PR: MAP
T-HbO_2_ (%)	0.429 (*p* < 0.001)	− 0.477 (*p* < 0.001)	0.513 (*p* < 0.001)
RBC tissue fraction (%)	0.402 (*p* < 0.001)	− 0.504 (*p* < 0.001)	0.535 (*p* < 0.001)
Total perfusion (% RBC x mm/s)	0.359 (*p* < 0.001)	− 0.576 (*p* < 0.001)	0.608 (*p* < 0.001)
Speed resolved perfusion < 1 mm/s	0.424 (*p* < 0.001)	− 0.578 (*p* < 0.001)	0.600 (*p* < 0.001)
Speed resolved perfusion 1–10 mm/s	0.169 (*p* = 0.171)	− 0.564 (*p* < 0.001)	0.538 (*p* < 0.001)
Speed resolved perfusion > 10 mm/s	0.267 (*p* = 0.066)	− 0.259 (*p* = 0.056)	0.324 (*p* = 0.012)
Laser-Doppler Flow (PU)	0.319 (*p* = 0.012)	− 0.439 (*p* < 0.001)	0.484 (*p* < 0.001)

No significant differences between treatments were observed in SpO_2_ or ƒR (Supplementary Table 1).

## Discussion

Overall cutaneous microcirculation was better with the addition of vatinoxan in male Wistar rats sedated with medetomidine and midazolam. In addition, PR was significantly higher and MAP significantly lower in the vatinoxan-treated groups. No hypotension was observed. The results are in accordance with previous findings in rats [20]. Fentanyl did not significantly affect any of the systemic or microcirculatory variables. The negative correlation between MAP and the slow microcirculatory flow speeds demonstrates that increased blood pressure led to decreased cutaneous capillary perfusion. The association was strengthened with the PR:MAP ratio, highlighting the hemodynamic interaction between vasoconstriction and bradycardia. By preventing the initial vasoconstriction in the vascular smooth muscle, vatinoxan promoted better microcirculation despite lower MAP values in rats sedated with the medetomidine-based protocols tested in this study. While the effect appeared consistent through the varying indicators of cutaneous oxygen delivery, the a priori power analysis for this study did not account for the larger variation in the presence of vatinoxan. Consequently, the null hypothesis of no significant effect was retained for a number of comparisons.

Given that microcirculatory parameters are sensitive indicators of tissue-level oxygen delivery, potential ventilatory effects of fentanyl warrant consideration. The combination of medetomidine, midazolam and fentanyl has been previously shown to produce surgical anesthesia in rats and is suitable for repeated use [32]. However, opioids such as fentanyl can decrease alveolar ventilation [33–35] and thereby it can reduce arterial oxygen content [36]. Consistent with this, in spontaneously ventilating rats pre-medicated with dexmedetomidine, the administration of alfentanil has been shown to decrease blood pH and PaO₂ while increasing PaCO₂ [37]. Similarily, administration of medetomidine and midazolam combined with butorphanol, an opioid antagonist-agonist, has been reported to induce respiratory depression and alter SpO_2_ and arterial oxygen content in both rats and mice [38–40]. In animals breathing room air, hypoventilation would be expected to manifest as a fall in SpO₂. A meaningful reduction in SpO₂ would, in turn, lower arterial oxygen content, promoting a corresponding desaturation within the microcirculation as indicated by a reduction in T-HbO₂. However, the addition of fentanyl did not have a detectable additive effect on ƒR, SpO₂ or T-HbO_2_ in the present study.

Moreover, while SpO₂ values were uniformly low across treatments, with median peripheral oxygen saturation ranging from 84 to 94%, SpO₂ did not correlate with T-HbO₂ or ƒR. Systemic hypoxemia would be expected to have a negative impact on peripheral tissue oxygenation, unless the rate-limiting step of oxygen delivery was mainly affected by differences in capillary perfusion. However, the absence of arterial blood gas measurements and previous methodological validation in rats should be taken into consideration when construing the present results. Further efforts on the effects of fentanyl on CO_2_ retention and pulmonary gas exchange in rats sedated with medetomidine-based protocols are warranted. Regardless, the unequivocally low SpO_2_ represents an important welfare implication during rat sedation. The findings strongly support the routine provision of supplemental oxygen with these protocols (MM/MMF) to avoid hypoxia, particularly of organs that are most sensitive to oxygen-deprivation [41].

When administered alone, fentanyl also markedly decreases heart rate in rats [42]. Moreover, when rats are anesthetized with pentobarbital, fentanyl also significantly reduces MAP [43]. Further, the administration of alfentanil produced significant decrease in both PR and MAP in rats pre-treated with dexmedetomidine [38]. In the present study, however, no significant differences in PR or MAP were observed with the addition of fentanyl, and the modulatory effects of vatinoxan were equally sustained. The outcome is interesting as it suggests that in medetomidine-treated rats, the tested moderate dose of fentanyl is void of any marked cardiovascular impact. While it is possible that a fentanyl-induced decrease in PR and/or MAP was offset by the stimulatory impact of a coinciding respiratory acidosis [44], marked hypoventilation was unlikely as no impact of fentanyl on SpO_2_ was detected. Notably, both PR and MAP remained closer to normal reference values of awake rats [45] with the addition of vatinoxan at nearly all time points, regardless of the presence of fentanyl.

The main limitations of this study are related to the technology used for assessing microcirculation. The PeriFlux 6000 EPOS system is novel and not yet validated for use in rodents. While this system is validated for human application, particularly in assessing cutaneous tissue perfusion in patients with peripheral arterial disease [46] and diabetes [47], its accuracy and reliability in animal models remain unverified. As such, while the data provide valuable insights into relative changes in microcirculation between treatment groups, caution should be exercised when interpreting absolute values or drawing direct comparisons to human data. While the study appeared adequately powered in detecting treatment effects in both PR and MAP, the sample size proved too small to detect moderate differences in most microcirculatory parameters. This was primarily due to the high variability observed in microcirculatory parameters in rats receiving vatinoxan, despite carefully controlled study settings. In addition, statistical power was slightly reduced due to the exclusion of one animal from the MMV group. Nevertheless, by considering the observed treatment medians, the positive effect of vatinoxan appeared consistent and unidirectional across the investigated treatments. Based on the present results, it will also be possible to make more accurate sample size estimations for the applied methodology.

The use of single-sex subjects represents a further limitation, as current research standards emphasize the importance of including both sexes to assess potential sex-related differences [48]. Future studies should also include female subjects to determine whether the observed effects of these drugs are consistent across sexes.

## Conclusions

Vatinoxan improved cutaneous microcirculation and oxygenation parameters, most likely by attenuating the peripheral vasoconstrictive and bradycardic effects of medetomidine in rats. This outcome represents a potential refinement of α₂-agonist-based sedation protocols for this species.

## Supplementary Information

Below is the link to the electronic supplementary material.

Supplementary Material 1.

## Data Availability

The data that support the findings of this study are available from the corresponding author upon reasonable request.
