# Supplementary material for: Microcirculatory impact of vatinoxan and fentanyl in male Wistar rats sedated with medetomidine and midazolam
Source: BMC Vet Res. 2026 Apr 21;22:326. doi: 10.1186/s12917-026-05483-y (PMC13235014; doi:10.1186/s12917-026-05483-y)
Supplement: Supplementary file 1 — Supplementary Material 1. [file 12917_2026_5483_MOESM1_ESM.docx]

**Supplementary Table 1.**

| SpO_2_ (%) | Treatment | Time (minutes) | 10 | 20 | 30 | 40 | 60 |
| --- | --- | --- | --- | --- | --- | --- | --- |
|  | MM | N | 3 | 7 | 8 | 8 | 8 |
|  |  | Median (range) | 89 (88–97) | 89 (83–100) | 88 (84–91) | 86 (83–89) | 85 (84–90) |
|  | MMF | N | 4 | 8 | 8 | 8 | 8 |
|  |  | Median (range) | 93 (83–96) | 88 (78–95) | 86 (77–96) | 86 (75–90) | 84 (78–87) |
|  | MMV | N | 6 | 7 | 7 | 7 | 7 |
|  |  | Median (range) | 91 (87–93) | 90 (85–95) | 90 (82–90) | 89 (84–92) | 88 (83–93) |
|  | MMVF | N | 7 | 8 | 8 | 8 | 8 |
|  |  | Median (range) | 94 (89–95) | 91 (82–98) | 91 (72–100) | 86 (79–92) | 89 (80–92) |
| ƒR (breaths per minute) | Treatment | Time (minutes) | 10 | 20 | 30 | 40 | 60 |
|  | MM | N | 3 | 7 | 7 | 8 | 7 |
|  |  | Median (range) | 66 (60–78) | 78 (60–96) | 78 (60–96) | 78 (66–90) | 78 (66–102) |
|  | MMF | N | 4 | 8 | 8 | 8 | 8 |
|  |  | Median (range) | 84 (60–90) | 66 (60–90) | 75 (60–90) | 69 (60–78) | 75 (66–84) |
|  | MMV | N | 6 | 7 | 7 | 7 | 7 |
|  |  | Median (range) | 78 (60–84) | 78 (66–84) | 72 (60–84) | 72 (66–78) | 72 (66–78) |
|  | MMVF | N | 7 | 8 | 8 | 8 | 8 |
|  |  | Median (range) | 66 (60–72) | 66 (60–78) | 66 (54–72) | 66 (54–72) | 66 (60–84) |

**Supplementary Table 1.** SpO_2_ and ƒR of rats administered with medetomidine 0.25 mg/kg and midazolam HCl 2 mg/kg (MM) N=8, MM + vatinoxan HCl 5 mg/kg (MMV) N=7, MM + fentanyl HCl 0.01 mg/kg (MMF) N= 8 or MMF + vatinoxan HCl 5 mg/kg (MMVF) N=8.
